# A Novel Bacterium-Like Particle-Based Vaccine Displaying the SUDV Glycoprotein Induces Potent Humoral and Cellular Immune Responses in Mice

**DOI:** 10.3390/v11121149

**Published:** 2019-12-11

**Authors:** Shengnan Xu, Cuicui Jiao, Hongli Jin, Wujian Li, Entao Li, Zengguo Cao, Zhikang Shi, Feihu Yan, Shengnan Zhang, Hongbin He, Hang Chi, Na Feng, Yongkun Zhao, Yuwei Gao, Songtao Yang, Jianzhong Wang, Hualei Wang, Xianzhu Xia

**Affiliations:** 1 College of Animal Science and Technology, Jilin Agricultural University, Changchun 130118, China; xsn881222@163.com (S.X.); s12k20180101@163.com (Z.S.); 2 Key Laboratory of Jilin Province for Zoonosis Prevention and Control, Military Veterinary Research Institute, Academy of Military Medical Sciences, Changchun 130122, China; jcc1990512@163.com (C.J.); jin8616771@163.com (H.J.); lwj636567@163.com (W.L.); liet0706@163.com (E.L.); cengcao@hotmail.com (Z.C.); yanfh1990@gmail.com (F.Y.); Zhang_Shengnan1992@163.com (S.Z.); ch_amms@163.com (H.C.); fengna0308@126.com (N.F.); zhaoyongkun1976@126.com (Y.Z.); gaoyuwei@gmail.com (Y.G.); yst62041@163.com (S.Y.); whl831125@163.com (H.W.); 3 Key Laboratory of Zoonosis Research, Ministry of Education, College of Veterinary Medicine, Jilin University, Changchun 130062, China; 4 College of Veterinary Medicine, South China Agricultural University, Guangzhou 510642, China; 5 College of Wildlife and Protected Area, Northeast Forestry University, Harbin 150040, China; 6 Key Laboratory of Animal Resistant Biology of Shandong, Ruminant Diseases Research Center, College of Life Sciences, Shandong Normal University, Jinan 250014, China; hongbinhe@sdnu.edu.cn; 7 Jiangsu Co-innovation Center for Prevention and Control of Important Animal Infectious Diseases and Zoonoses, Yangzhou 225000, China

**Keywords:** SUDV, subunit vaccine, bacterium-like particles, eGP, immune response

## Abstract

Sudan virus (SUDV) causes severe lethal hemorrhagic fever in humans and nonhuman primates. The most effective and economical way to protect against Sudan ebolavirus disease is prophylactic vaccination. However, there are no licensed vaccines to prevent SUDV infections. In this study, a bacterium-like particle (BLP)-based vaccine displaying the extracellular domain of the SUDV glycoprotein (eGP) was developed based on a gram-positive enhancer matrix-protein anchor (GEM-PA) surface display system. Expression of the recombinant GEM-displayed eGP (eGP-PA-GEM) was verified by Western blotting and immunofluorescence assays. The SUDV BLPs (SBLPs), which were mixed with Montanide ISA 201VG plus Poly (I:C) combined adjuvant, could induce high SUDV GP-specific IgG titers of up to 1:40,960 and robust virus-neutralizing antibody titers reached 1:460. The SBLP also elicited T-helper 1 (Th1) and T-helper 2 (Th2) cell-mediated immunity. These data indicate that the SBLP subunit vaccine has the potential to be developed into a promising candidate vaccine against SUDV infections.

## 1. Introduction

Ebolaviruses, members of the family Filoviridae, cause severe hemorrhagic fever in primates and respiratory disease in pigs, and induce high morbidity and mortality [1,2,3]. The 2014 outbreak in Western Africa was the largest Ebola virus disease (EVD) outbreak in history, resulting in 11,291 deaths and receiving unprecedented attention worldwide [4]. Unfortunately, an EVD outbreak occurred again in 2018 in eastern Democratic Republic of Congo and, as of August 2019, a total of 2997 EVD cases had been reported, including 2892 confirmed cases with 1998 deaths (overall case fatality rate of 67%) [5]. Since the first outbreak was reported in 1976, five different species of ebolaviruses, including Ebola virus (EBOV), Sudan virus (SUDV), Reston virus (RESTV), Bundibugyo virus (BDBV), and Taï Forest virus (TAFV,) have been identified and their genomic sequences differ by ~35–45% [2]. Excluding EBOV, SUDV has the highest mortality and outbreak rates among the species of ebolaviruses. SUDV has emerged at least six times with average mortality rates of up to 53.76% [6,7]. Multiple vaccines and monoclonal antibodies against EBOV have been developed, but very few of them can effectively prevent and control SUDV infection [8]. Therefore, there is an urgent need to develop a safe and efficacious vaccine against SUDV.

The EBOV genome encodes nine structural proteins, including the surface envelope glycoprotein (GP) as the only membrane protein [9]. The mature GP protein forms homotrimers on the surface of infected cells and virions, which is crucial for receptor binding, viral entry, and host immunity induction, making GP an ideal vaccine target [10]. A number of candidate vaccines for EBOV have demonstrated protection against lethal EBOV challenge in animal models and progressed to clinical trials, and most of these vaccines, including DNA vaccines [11], vaccines based on viral vectors, such as recombinant adenovirus [12] and vesicular stomatitis virus (VSV) [13], and protein-based vaccines, such as virus-like particles (VLPs) [14], have been based on GP. Most notably, a number of the candidate vaccines currently under clinical phase evaluation are viral vector-based vaccines [15,16]. While recombinant vesicular stomatitis virus (rVSV)-based and recombinant adenovirus type-5 vector (rAd5)-based EBOV candidate vaccines have been shown to be highly efficacious against EBOV infection and transmission, numerous side effects, such as fever, acute arthritis, and skin lesions, have been reported [15,17,18]. In addition, preexisting antibodies, costs, and side effects should also be considered. In contrast, EBOV subunit vaccines may offer a relatively safe alternative for inducing immune responses against an antigen of interest [19,20]. However, EBOV subunit vaccines would face hurdles related to immunogenicity, thus requiring selection of the antigen delivery vector and an appropriate adjuvant or even adjuvant combinations [15].

Displaying heterologous pathogen-derived proteins on the surface of bacteria is an effective and safe way to enhance the immunogenicity of a vaccine [21]. Gram-positive enhancer matrix-protein anchor (GEM-PA), a novel surface display system, has a wide range of biotechnological applications, especially in the development of vaccine delivery systems [21]. The GEM-PA surface display system consist of GEM particles based on nonliving and nongenetically modified gram-positive *Lactococcus lactis* (*L. lactis*) bacteria and a PA derived from the *L. lactis* peptidoglycan hydrolase AcmA [22]. Antigens fused with the PA can be anchored effectively and stably to the peptidoglycan of GEM particles and induce antigen-specific immune responses [23]. Furthermore, a vaccine approach based on the GEM-PA surface display system eliminates the risk of including recombinant DNA in the vaccine [24,25]. As a safe, effective, inexpensive, multifunctional platform with a high loading capacity for protein antigens, the GEM-PA surface display system has been applied to a Middle East respiratory syndrome-related coronavirus vaccine [26], a respiratory syncytial virus vaccine, and porcine circovirus type 2 vaccines [25], among others.

In this study, we developed a novel bacterium-like particle (BLP) vaccine displaying the SUDV glycoprotein by using the GEM-PA surface display system.

## 2. Materials and Methods

### 2.1. Construction and Expression of Recombinant Baculoviruses

A novel microconsensus (Con) SUDVGP construct was designed through Weblogo, a web-based application. PA gene sequences were obtained from GenBank (GenBank: U17696.1, corresponding to nucleotides 904–1488). All of the genes were codon-optimized for the highest possible expression levels in insect cells and biochemically synthesized (Sangon Biotech, Shanghai, China). The eGP-PA fusion gene was amplified by PCR using synthetic oligonucleotide primers as listed in Table 1 and cloned into *Xba*I- and *Kpn*I-linearized pFastBac1 vector by ClonExpress Ultra One Step Cloning Kit (Vazyme Biotech). PFastBac1-eGP-PA was then transformed into *Escherichia coli* DH10Bac competent cells to generate recombinant bacmids. *Spodoptera frugiperda* (Sf9; Gibco, Grand Island, NY, USA) insect cells were transfected with the recombinant bacmids using Cellfectin II Reagent following the Bac-to-Bac Expression Systems manual (Invitroge, Waltham, MA, USA). Recombinant baculoviruses (rBV-eGP-PA) were harvested at 5 days post transfection and defined as the first passage 1 (P1) premaster virus. These viruses were expanded in Sf9 cells to generate virus stocks.

### 2.2. Expression Analyses of Recombinant Baculoviruses

For indirect immunofluorescence assay (IFA) analysis, Sf9 cells were grown in 24-well plates and infected with rBV-eGP-PA for 48 h and then fixed with 80% cold acetone for 30 min at room temperature (RT). Subsequently, the cells were incubated with a 1:500 dilution of a mouse anti-SUDV-GP1 monoclonal antibody (prepared and stored in our laboratory; only reacts specifically with SUDV GP) in a buffer containing 1% bovine serum albumin (BSA) for 1 h at RT. After three washes with phosphate buffered saline (PBS) containing 0.05% Tween 20 (PBST), a 1:200 dilution of a fluorescein isothiocyanate (FITC)-labeled goat anti-mouse IgG antibody (Sigma, St. Louis, MO, USA) was added with 0.3% Evans blue for 1 h at RT. The cells were observed with a fluorescence microscope after washing. 

For Western blotting (WB), expressed fusion protein (eGP-PA) was separated by 10% SDS-PAGE under denaturing conditions, and the proteins were transferred to a nitrocellulose (NC) membrane (GE Healthcare Life Sciences, Freiburg, Germany) for immunoblot analysis with the mouse anti-SUDV-GP1 monoclonal antibody. Detection was then performed with a horseradish peroxide (HRP)-conjugated goat anti-mouse antibody and enhanced chemiluminescence. 

### 2.3. Preparation of SUDV BLP (SBLP) Vaccine

The preparation of GEM particles has been described in detail elsewhere [26]. Briefly, *L. lactis* MG1363 cells were cultured in M17 broth (Oxoid) supplemented with 0.5% glucose at 30 °C. GEM particles were obtained by boiling harvested *L. lactis* in 10% trichloroacetic acid (TCA) for 30 min, followed by extensive washing with PBS. One unit (U) was defined as 2.5 × 10^9^ GEM particles. Finally, the GEM particles were resuspended in PBS and stored at −80 °C until use. 

Preparation of the GEM-based vaccine was conducted as follow: supernatants, following supersonic schizolysis containing the eGP-PA fusion protein, were mixed with GEM particles for 30 min at RT. After binding, the eGP-PA-GEM complexes were collected, washed five times with sterile PBS, and resuspended in PBS to produce SBLP, which were the GEM particles displaying the eGP antigen on their surface. The target was determined by using a GP-specific antibody for WB. The amount of bound eGP-PA was compared to BSA standards by analysis of the SDS-PAGE results using software Quantity One. 

### 2.4. Identification of GEM Particle Binding

For the SDS-PAGE and WB analyses of the GEM particles, the eGP-PA-GEM complexes were treated with 5× SDS loading buffer for 10 min at 100 °C, separated using 10% SDS-PAGE gel, and then transferred onto a nitrocellulose (NC) membrane for WB analysis with the mouse anti-SUDV-GP1 monoclonal antibody. For IFA analysis, GEM particles with bound eGP-PA were blocked with 3% BSA for 30 min at 37 °C. Then, incubations with the primary antibody (mouse anti-SUDV-GP1 monoclonal antibody) and secondary antibody (FITC-labeled goat anti-mouse IgG) were performed as previously described (Section 2.2), and the particles were viewed and imaged using a Zeiss microscope with incident UV illumination and a Zeiss Axiovision digital imaging system (Zeiss, Oberkochen, Germany).

### 2.5. Immunizations of Mice and the Associated Ethics Statement

In total, two batches of BALB/c mice (six- to eight-weeks-old females) were purchased from the Changchun Institute of Biological Products Co., Ltd. (Changchun, China) and immunized. Poly (I:C) (Sigma, USA), aluminum hydroxide (Alum; Thermo, USA), and Montanide ISA 201VG (ISA 201VG; Seppic, France) were purchased. All research was in compliance with the Welfare and Ethics of Laboratory Animals of China (GB 14925-2001), and protocols were approved by the Animal Welfare and Ethics Committee of the Veterinary Institute at the Academy of Military Medical Sciences (JSY-DW-2018-02). 

In batch I, mice were randomly divided into 6 groups and immunized as shown in Table 2. In batch II, mice were randomly divided into 3 groups and then vaccinated with 10 μg eGP-PA-GEM alone or with ISA 201VG plus Poly (I:C) compound adjuvant. In the two batches of animal experiments, all of the mice in the control group received both the same volume of PBS at the same time points. Immunizations were performed on study days 0 and 21. Blood samples were collected at two, four, and five weeks post immunization. 

### 2.6. Analysis of Mouse Antibody Titers by Indirect ELISA

Immune sera were collected and analyzed for SUDV-specific antibodies by indirect enzyme-linked immunosorbent assay (ELISA). Briefly, the assays were performed in 96-well polystyrene microtiter plates (Corning Costar, USA) that were precoated with purified GP proteins at a concentration of 2 μg/mL overnight at 4 °C and blocked for 1.5 h at 37 °C. Serial dilutions of the serum samples were incubated at 37 °C for 1.5 h, and the secondary antibodies included HRP-conjugated goat anti-mouse IgG, HRP-conjugated IgG1, and HRP-conjugated IgG2a, which were incubated at 37 °C for 1 h. To develop the colorimetric reaction, the substrate tetramethylbenzidine (Sigma, USA) was added to each well and then the reaction was stopped with 2 M H_2_SO_4_. The absorbance was read at 450 nm. Titers were determined as the highest dilution at which the mean absorbance of the sample was 2-fold greater than the mean absorbance of the same dilution of control serum.

### 2.7. Pseudovirion Neutralization Assay

The neutralizing activity of sera from vaccinated mice against pseudoviruses containing an SUDV GP, based on a human immunodeficiency virus backbone, was analyzed as described in previous studies [27]. Briefly, diluted serum samples were added to Huh7 cells, followed by the addition of 100× 50% tissue-culture infective dose (TCID_50_) of pseudotype virus (prepared in a volume equal to that of the serum samples), which were incubated at 37 °C for 1 h before addition to the Huh7 cells. After a 5 h incubation, the medium was replaced with DMEM containing 10% FBS. The plates were incubated for 48 h at 37 °C and luciferase activity was measured using Infinite M200. Neutralizing activity is expressed as the percentage reduction in luciferase activity between the sample wells and control wells: ((luciferase activity in the control well – luciferase activity in the sample well)/(luciferase activity in the control well)) × 100%. The 50% neutralization dose (ND_50_) was calculated using GraphPad Prism.

### 2.8. IFN-γ, IL-4, and TNF-α ELISpot Assays

Splenocytes were isolated from vaccinated mice at 8 days after the second vaccination. The cells were cultured in Roswell Park Memorial Institute (RPMI) 1640 medium (Gibco, San Diego, CA, USA) containing 10% FBS and then stimulated with or without purified SUDV GP antigen (10 μg/mL). Following an incubation at 37 °C in 5% CO2 for 36 h, the splenocytes producing interferon-gamma (IFN-γ), interleukin 4 (IL-4) or tumor necrosis factor alpha (TNF-α) were measured using mouse enzyme-linked immunospot (ELISpot) kits (Mouse IFN-γ/IL-4 ELISpot kit, Mabtech AB, Stockholm, Sweden) according to the manufacturer’s instructions. The spot-forming cells (SFCs) were counted using an automated ELISpot reader (AID ELISPOT reader-iSpot, AID GmbH, GER).

### 2.9. ELISA Measurement of Cytokine Secretion

To detect the levels of the cytokines interleukin 2 (IL-2), IL-4, interleukin 10 (IL-10), IFN-γ, and TNF-α, splenocytes were isolated from vaccinated mice at 8 days after the second vaccination and then cultured (1 × 10^6^ cells/mL) with stimulation as described above. After 48 h, the supernatants of the stimulated cells were evaluated using murine IL-2, IL-4, IL-10, IFN-γ, and TNF-α ELISA kits (Mabtech AB, Sweden) according to the manufacturer’s instructions. 

### 2.10. Statistical Analysis

Statistical analysis was performed using GraphPad Prism software (GraphPad Software, La Jolla, CA, USA). Significant differences between two means were determined by an unpaired Student’s *t*-test. Data are presented as the mean ± standard error unless otherwise indicated. Statistical significance is indicated as * *p* < 0.05, ** *p* < 0.01, *** *p* < 0.001, and **** *p* < 0.0001.

## 3. Results

### 3.1. Expression of SUDV eGP-PA Fusion Protein

**Figure 1:**
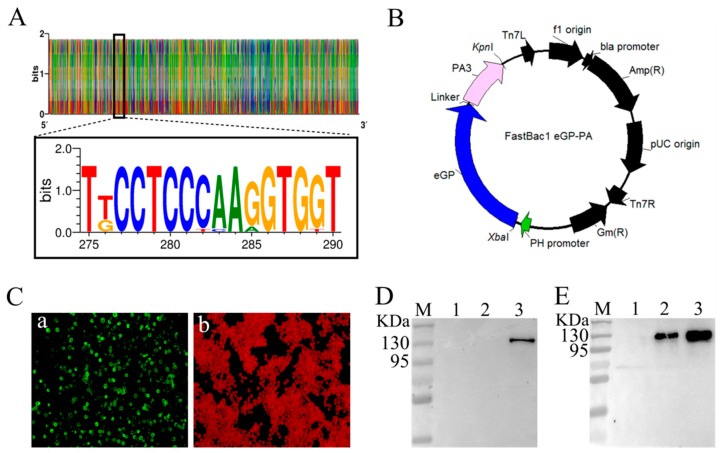
Construction and detection of a recombinant baculovirus. (**A**) Eighteen SUDVsequence logo of differences and preference characteristics. (**B**) Schematic of the recombinant baculovirus expressing the SUDV eGP-PA fusion protein. (**C**) IFA detection of eGP-PA expression in baculovirus-infected Sf9 insect cells (magnification of microscopy images, 200×). Cells were infected with the recombinant baculoviruses in (a) and were mock infected in (b). (**D**) WB analysis of antibody specificity. M: molecular weight marker; Lane 1: Marburg virus (MARV) virus-like particles; Lane 2: EBOV bacterium-like particles; Lane 3: recombinant baculovirus (rBV-eGP-PA)-infected Sf9 cell lysate. (**E**) WB analysis of eGP-PA fusion protein expression format. M: molecular weight marker; Lane 1: culture medium supernatant; Lane 2: precipitate following supersonic schizolysis; Lane 3: supernatant following supersonic schizolysis.

As shown in Figure 1A, according to sequence preference characteristics, we designed a new synthetic microconsensus GP based on all strains of SUDV from 1976–2012 that were available on the National Center for Biotechnology Information (NCBI) website. The strategy for designing the eGP-PA fusion protein, in which eGP is fused to the PA with a linker, is shown in Figure 1B. IFA results showed that, compared to control cells, Sf9 cells expressing the eGP-PA fusion protein emitted strong green fluorescence (Figure 1C). WB showed that eGP-PA was successfully expressed and present as a 130 KDa protein in supernatants and precipitates after supersonic schizolysis (Figure 1D,E). Furthermore, as shown in Figure 1D, the mouse anti-SUDV-GP1 monoclonal antibody only reacted specifically with SBLP. All these results indicated that the recombinant eGP-PA protein reacted with anti-SUDV-GP1 monoclonal antibodies with good antigenicity (Figure 1C–E). 

### 3.2. Location of the Fusion Protein on GEM Particles

**Figure 2:**
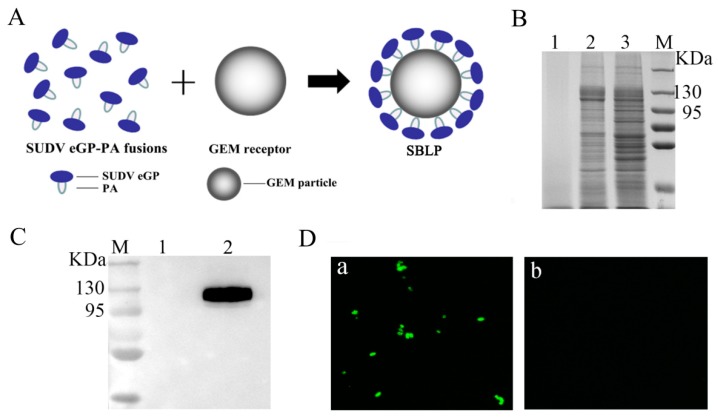
Characterization of eGP-PA binding with GEM particles. (**A**) Schematic diagram of the production of SBLP. The eGP fragment of interest was fused at its C terminus with the LysM motif (PA). The PA bound noncovalently to the peptidoglycan of BLPs. (**B**) SDS-PAGE analysis of the binding of eGP-PA fusion protein with GEM particles. M: molecular weight marker; Lane 1: GEM particles; Lane 2: recombinant eGP-PA fusion protein bound to GEM particles; Lane 3: recombinant eGP-PA fusion protein. (**C**) WB analysis of the binding of eGP-PA fusion protein with GEM particles. M: molecular weight marker; Lane 1: GEM particles; Lane 2: recombinant eGP-PA fusion protein bound to GEM particles. (**D**) IFA analysis of the binding of the eGP-PA fusion protein with GEM particles (magnification of microscopy images, 1000×). a: eGP-PA-GEM complexes. b: GEM particles.

The production process for SBLP is shown in Figure 2A. A cell-free extract containing eGP-PA was mixed with GEM particles, and, after washing, the particles were subjected to SDS-PAGE analysis (Figure 2B). Analysis by Quantity One indicated that 1 U of GEM particles could bind approximately 65.2 μg of the eGP-PA fusion protein (data not shown). The surface location of eGP-PA on the GEM particles was also analyzed by WB (Figure 2C) and IFA (Figure 2D) with anti-SUDV-GP1 monoclonal antibody. The WB results showed that the eGP-PA fusion protein was bound to GEM particles. Moreover, microscopic observation indicated that, compared with the GEM particles alone, the combination of the GEM particles and eGP-PA fusion protein emitted strong green fluorescence. Therefore, the above results indicated that the eGP-PA fusion protein was anchored to GEM particles.

### 3.3. Antibody Responses Induced by SBLP

**Figure 3:**
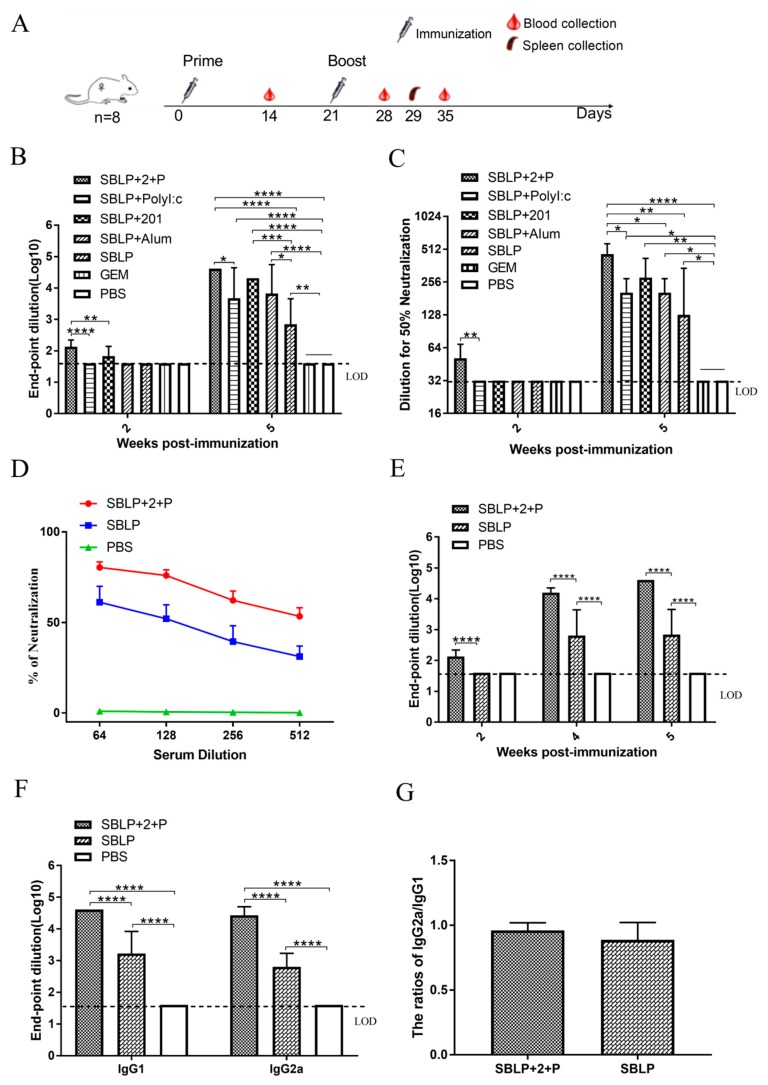
Serum antibody responses induced by SUDV SBLP. Serum samples were collected by retro-orbital plexus puncture at weeks 2, 4, and 5. EBOV GP-specific total IgG, IgG1, and IgG2a antibody responses were measured by indirect ELISA with the purified GP protein and are displayed as the end-point dilution titers. The horizontal dotted line in the figure indicates the limit of detection (LOD). *n* = 8 mice/group/time point. Data are shown as the mean ± SD and were analyzed by one-way ANOVA. * *p* < 0.05, ** *p* < 0.01, *** *p* < 0.001, **** *p* < 0.0001. (**A**) Schematic of the experiment. (**B,C**) Analysis of serum antibody titers induced by different adjuvants by ELISA and neutralization of SUDV GP-pseudotyped virus. Neutralizing antibody titers were measured with Huh7 cells and 100× TCID_50_ of pseudotyped virus. (**D**) Neutralization of the SUDV GP-pseudotyped virus. Serum samples were collected at 2 weeks after the second immunization. (**E**) Total anti-SUDV IgG antibody titers of SBLP with ISA 201VG plus Poly (I/C) and SBLP immunized mice at weeks 2, 4, and 5. (**F**) Serum anti-SUDV antibody subclass responses detected at 2 weeks after the second immunization. (**G**) Ratios of IgG2a/IgG1.

To select the most effective adjuvant from among ISA 201VG plus Poly (I:C), Poly (I:C), Alum, and ISA 201VG for SBLP immunization, 2 weeks after immunization, sera samples were obtained and analyzed (Figure 3A). The results showed that all mice immunized with SBLP had significantly stronger SUDV GP-specific IgG and neutralizing antibody titers than mice immunized with PBS or GEM. In particular, the SBLP + 2 + P group had significantly higher anti-SUDV GP IgG titers than the SBLP alone group (*p* < 0.0001) (Figure 3B). Furthermore, when the sera samples from vaccinated mice were tested for neutralizing activity, compared with other adjuvant groups, the group immunized with ISA 201VG plus Poly (I:C) as an adjuvant had greater neutralization across the most dilutions. (Figure 3B,C). Therefore, the ISA 201VG plus Poly (I:C) compound adjuvant was selected for further vaccination experiments in mice.

Then, we further compared the antibody responses against SUDV GP induced by SBLP with ISA 201VG plus Poly (I:C) and those induced by SBLP. Pseudotyped virus neutralization assay data showed that the SBLP + 2 + P group exhibited stronger neutralizing antibodies than the SBLP group (Figure 3D). ELISA results showed that sera from mice immunized with SBLP with ISA 201VG plus Poly (I:C) strongly reacted with the SUDV GP protein after receiving a second immunization, reaching endpoint titers of up to 1:40,960 (Figure 3E). By contrast, the antibody levels in sera from SBLP immunized mice showed no significant differences between the samples harvested at 1 week or 2 weeks after the second immunization, indicating that the antibody response plateaued (Figure 3E). A similar phenomenon was also found for sera SUDV GP-specific IgG1 (Figure 3F) and IgG2a (Figure 3F) antibodies at 5 weeks post immunization in mice immunized with SBLP with ISA 201VG plus Poly (I:C) and SBLP. Interestingly, the ratios of IgG2a/IgG1 of SBLP with ISA 201VG plus Poly (I:C) were no significant differences than those of SBLP (Figure 3G), indicating that the SBLP induced a mixed T-helper 1 (Th1) and T-helper 2 (Th2) immune responses.

### 3.4. Antigen-Specific Cellular Immune Responses

**Figure 4:**
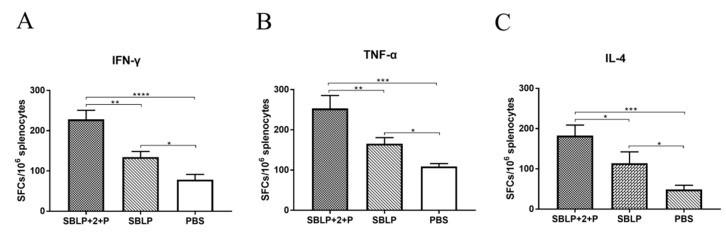
ELISpot analysis of IFN-γ, IL-4, and TNF-α secretion by mouse splenocytes. The splenocytes were collected from each group 8 days after the second immunization treated and analyzed. The secretion of (**A**) IFN-γ, (**B**) IL-4, and (**C**) TNF-α was measured by using ELISpot kit. Data are shown as the mean ± SD and were analyzed using one-way ANOVA (* *p* < 0.05, ** *p* < 0.01, *** *p* < 0.001).

After confirming that SBLP with ISA 201VG plus Poly (I:C) successfully induced an enhanced antibody responses in mice, we next evaluated the T-cell responses in mice following vaccination. The IFN-γ, IL-4, and TNF-α secretion by mouse splenocytes was measured in ELISpot assays. As shown in Figure 4, the SFCs indicative of IFN-γ, IL-4, or TNF-α production by splenocytes from mice immunized with SBLP with ISA 201VG plus Poly (I:C) were significantly more than those from mice immunized with SBLP, indicating that both the Th1 and Th2 arms of adaptive immunity were activated.

### 3.5. SBLP Vaccine-Enhanced Splenocyte Cytokine Secretion

**Figure 5:**
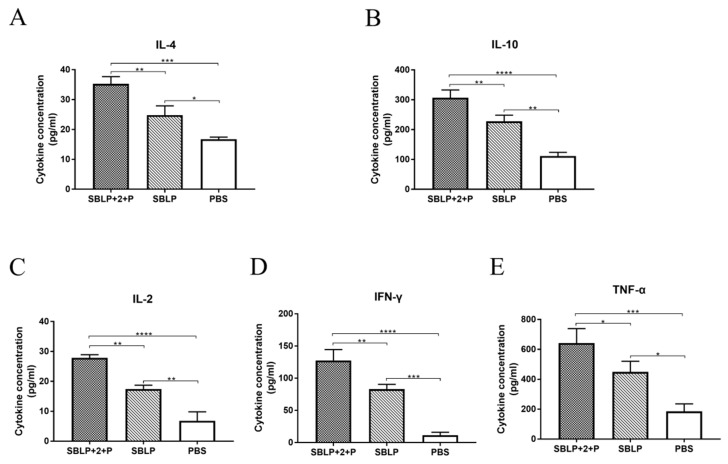
Quantities of IL-2, IL-4, IL-10, IFN-γ and TNF-α secreted by splenocytes. Splenocytes were prepared from 3 mice per group at 8 days after the second vaccination. Cell-free supernatants were harvested at 48 h after incubation and measured to determine the levels (pg/mL) of (**A**) IL-4, (**B**) IL-10, (**C**) IL-2, (**D**) IFN-γ, and (**E**) TNF-α via commercial ELISA kits. Data are shown as the mean ± SD and were analyzed using one-way ANOVA (* *p* < 0.05, ** *p* < 0.01, *** *p* < 0.001 and **** *p* < 0.0001).

To further investigate antigen-specific cellular immune responses, the cytokines secreted by splenocytes were assayed using commercial ELISA kits. The levels of the cytokine IL-2, IL-4, IL-10, IFN-γ, and TNF-α secreted by splenocytes from the mice in the SBLP with ISA 201VG plus Poly (I:C) or SBLP alone group were significantly higher than those secreted by splenocytes from the mice in the PBS group (Figure 5). The secretion of IFN-γ, TNF-α, and IL-2 was associated with a Th1 profile, whereas the secretion of IL-4 and IL-10 were associated with a Th2 immune response. These data demonstrated that the SBLP vaccine enhanced the secretion of both type 1 cytokines and type 2 cytokines in splenocytes, especially in the presence of adjuvants.

## 4. Discussion

The resurgence of the 2018 EVD epidemic in eastern DRC reminds us that there is still the threat of filovirus infection around the world. Subunit vaccines are a promising platform to prevent EBOV infection due to their relative safety, induction of effective immune responses, and available methods for high-level production [28]. GP is a promising candidate antigen for an EBOV protein vaccine. Currently, most EBOV vaccines target the viral GP antigen. Notably, previous studies have shown that subunit vaccines based on eGP were able to protect vaccinated mice against lethal EBOV challenge [29,30]. Bazzill, J. D. et al. [31] proved that a recombinant EBOV antigen (eGP) incorporated into lipid-based nanoparticles could efficiently generate germinal center B cells and polyfunctional T cells while eliciting robust neutralizing antibody responses. Furthermore, the absence of the signal sequence and transmembrane domain facilitated protein expression [10]. Considering the immune effect and protein expression, we chose the soluble extracellular domain of the GP protein as the target immunogen in this study. 

The novel exogenous antigen delivery system in this study was based on nonliving, nongenetically modified *L. lactis* cells designated GEM particles. *L. lactis* has a long history of use in foods and is recognized as safe [32]. These particles can bind to externally-added heterologous antigens by means of PA with a high loading capacity and high affinity [22]. In addition, chimeric anchor fusion proteins can efficiently, strongly, and selectively bind to GEM particles in culture medium at RT within a short time period without the need for additional purification steps [33]. It is easier to obtain purified antigens in a BLP-based vaccine than in a virus-like particle (VLP) vaccine [26]. Therefore, the cost of production for vaccines using the GEM-PA surface display system will be low. Based on the above advantages, the GEM-PA surface display system has been applied for the development of a variety of vaccines [21]. In the present study, we developed a novel SBLP vaccine by using the GEM-PA surface display system. We found that the SBLP were immunogenic, especially in promoting Th1- and Th2-type immune responses. However, some studies have shown that immunization with unadjuvanted GP nanoparticle vaccines by intramuscular (IM) injection induces IgG1 antibodies but not IgG2a antibodies against GP [27,34,35]. Therefore, we assumed that this outcome might be related to the presence of GEM particles, which can induce Th1-type immune responses when used as a vaccine adjuvant [36,37,38]. This skewing might be the result of an interaction with Toll-like receptor (TLR)-2 by the peptidoglycan present in the GEM particles as TLR-2 activation can shift the immune response toward a Th1-type response [39,40]. As far as we know, this is the first report that an eGP-based SUDV subunit vaccine can induce a mixed Th1/Th2 immune response without an adjuvant. The importance of a mixed Th1 and Th2 response in mediating protection against lethal EBOV infection has been demonstrated in several reports [41,42,43,44,45]. 

To further improve the immune effect of the SBLP vaccine, we used ISA 201VG mixed with Poly (I:C) as an adjuvant. ISA 201VG, which was developed for commercial products made with water-in-oil-in-water emulsions, can effectively improve immune response and protection [46]. Poly (I:C) has been demonstrated to be a potent adjuvant with the ability to enhance host innate and adaptive immune responses [36,47,48]. Immunization of mice with ISA 201VG mixed with Poly (I:C)-adjuvanted SBLP resulted in significant increases in SUDV GP-specific IgG, IgG1, and IgG2a and SUDV pseudotyped virus-neutralizing antibody titers, which are considered to be important correlates of protection [34,49]. We used the ratio of IgG1 to IgG2a as an indirect method to evaluate induced Th1- and Th2-type biases, respectively, in immune responses. Interestingly, the ratio of IgG2a/IgG1 showed no significant difference between SBLP alone and with an adjuvant. However, in most studies, ISA 201VG and Poly (I:C) adjuvants induced a Th1-polarized immune response [50,51]. We assumed that the presence of the GEM particles may change this bias. Further investigations are warranted to confirm the immunological adjuvant function of the GEM particles in the SBLP subunit vaccine.

EBOV vaccines should simultaneously stimulate the specific humoral and cellular immunity essential for effective vaccination [52,53,54]. The protective cellular immune response is associated with the production of several cytokines, including IFN-γ, TNF-α, IL-2, IL-4, and IL-10 [10,55]. Our results showed that SBLP resulted in significant increases in the secretion of these cytokines and that these levels also significantly increased in the presence of the ISA 201VG plus Poly (I:C) adjuvant. Our results further proved that SBLP can boost immune responses through Th1 and Th2 pathways, which are considered to be important correlates of protection [41,42,43,44,45]. 

In conclusion, we successfully constructed an SBLP vaccine displaying the eGP protein antigen in this study. Our results clearly demonstrated that SBLP with ISA 201VG plus Poly (I:C) had high immunogenicity and could elicit robust specific humoral and cellular immunity in vaccinated mice. While protective efficacy evaluation of SBLP vaccines in animal models must be performed in a future study, our results strongly support the potential of GEM-PA particles as a display and delivery system for subunit vaccine development. Additionally, efforts are underway to optimize the SBLP primary immune response effect. 

## Figures and Tables

**Table 1:** Sequences of the primers used in the present study.

Primer	Sequence (5’–3’)	Restriction Enzyme Site
eGP F ^1^	CTTACATCTATGCGGCCGCT*TCTAGA*ATGCTACTAGTAAATCAGTCACACCA	*Xba*I
Linker-eGP R	ACCAGAACCACCACCAGAACCACCACGCCAGCCAGTCCACCAATTATCAT	-
Linker-PA F ^2^	GGTGGTTCTGGTGGTGGTTCTGGTGATGGTGCTTCTTCAG	-
Linker-PA R ^1^	TAGTACTTCTCGACAAGCTT*GGTACC*TTACTTGATACGCAGGTATTGACCGATC	*Kpn*I

^1^ The sequences of restriction enzyme sites are underlined and italicized. ^2^ The middle linker (Gly-Gly-Ser-Gly) x2 base sequences are underlined.

**Table 2:** The mouse vaccination protocols.

Group	*n*	Immunization Route	Antigen	Adjuvant
SBLP + 2 + P	8	intramuscular	10 μg eGP-PA-GEM	201VG + Poly(I:C)
SBLP + 201	8	intramuscular	10 μg eGP-PA-GEM	201VG
SBLP + PolyI:C	8	intramuscular	10 μg eGP-PA-GEM	Poly (I:C)
SBLP + Alum	8	intramuscular	10 μg eGP-PA-GEM	Alum
SBLP	8	Intramuscular	10 μg eGP-PA-GEM	-
GEM	8	intramuscular	GEM	-
PBS	8	intramuscular	PBS	-
